# The transition from the female-like great calls to male calls during ontogeny in southern yellow-cheeked gibbon males (*Nomascus gabriellae*)

**DOI:** 10.1038/s41598-021-01648-x

**Published:** 2021-11-11

**Authors:** Michal Hradec, Gudrun Illmann, Luděk Bartoš, Petra Bolechová

**Affiliations:** 1 Department of Ethology and Companion Animal Science, Faculty of Agrobiology, Food and Natural Resources, Czech University of Life Sciences Prague, Kamýcká 129, 16521 Praha 6-Suchdol, Czech Republic; 2 Department of Ethology, Institute of Animal Science, Praha Uhříněves, Czech Republic

**Keywords:** Zoology, Animal behaviour

## Abstract

It is well known that gibbons emit a pattern of vocalizations, which is specific for species and sex. A previous study showed, however, that immature southern yellow-cheeked gibbon (*Nomascus gabriellae*) males produce only female-like great calls from 2.3 to 5.3 years of age in co-singing interactions with their mothers. To date, nothing is known about how the vocal repertoire of a male changes from the female-like call (great call) to the male call (staccato notes and multi-modulation phrase) during vocal ontogeny. The goal of this study was to describe the transition from the female-like great call to the male call and the ontogeny of the male call. We predicted that the transition from the female-like great call to the male-specific call and the development of the male call is a normal part of the aging proces. If this is the case, the following phenomena will occur: (a) female vocalization should no longer be produced with the mature form of the multi-modulation phrase and (b) all stages of the male vocalization should occur gradually as the young male ages. Young males regularly emit both female-like great calls and male-specific calls between the ages of 5.6 to 7.1 years. Once the young males reached 7.1 years of age, they emitted male calls exclusively, and they continued to do so until the end of the observation period (at 8.11 years of age). It was confirmed that the young males emitted only female-like great calls during periods when they produced non-mature forms of a multi-modulation phrase (Fm_0,1_—none or one frequency modulation in second notes). Furhermore, the decrease in the number of female-like great calls was attributed to the development of the mature form of the multi-modulation phrase (Fm_2_—two or more frequency modulation in second notes), which developed with age. We also confirmed that the multi-modulation phrase developed gradually, while the development of the staccato notes occurred in leaps. A multi-modulation phrase developed as the initial part of the male-specific call. It was evolved from a simpler to a more complex form as the maximum frequency and age of the young males increased. Staccato notes subsequently developed in certain young males. Possible explanations for such vocal ontogeny in young males are discussed in this work.

## Introduction

The development of species-specific vocalization in non-human primates has been studied in a variety of ways, e.g., by observing normal vocal development, acoustic deprivation, isolation or cross-fostering^1^.

Most of these studies have focused on vocal ontogeny in non-human primates, notably in bushbabies (the *Galaginae* family^2,3^), squirrel monkeys (the *Saimiri* genus^4,5^), marmosets and tamarins (the *Callitrichidae* family, e.g.^6–8^), and macaques (the *Macaca* genus, e.g.^9–12^). With respect to other species of non-human primates, including gibbons (the Hylobatidae family), vocal ontogeny has received little attention.

**Figure 1:**
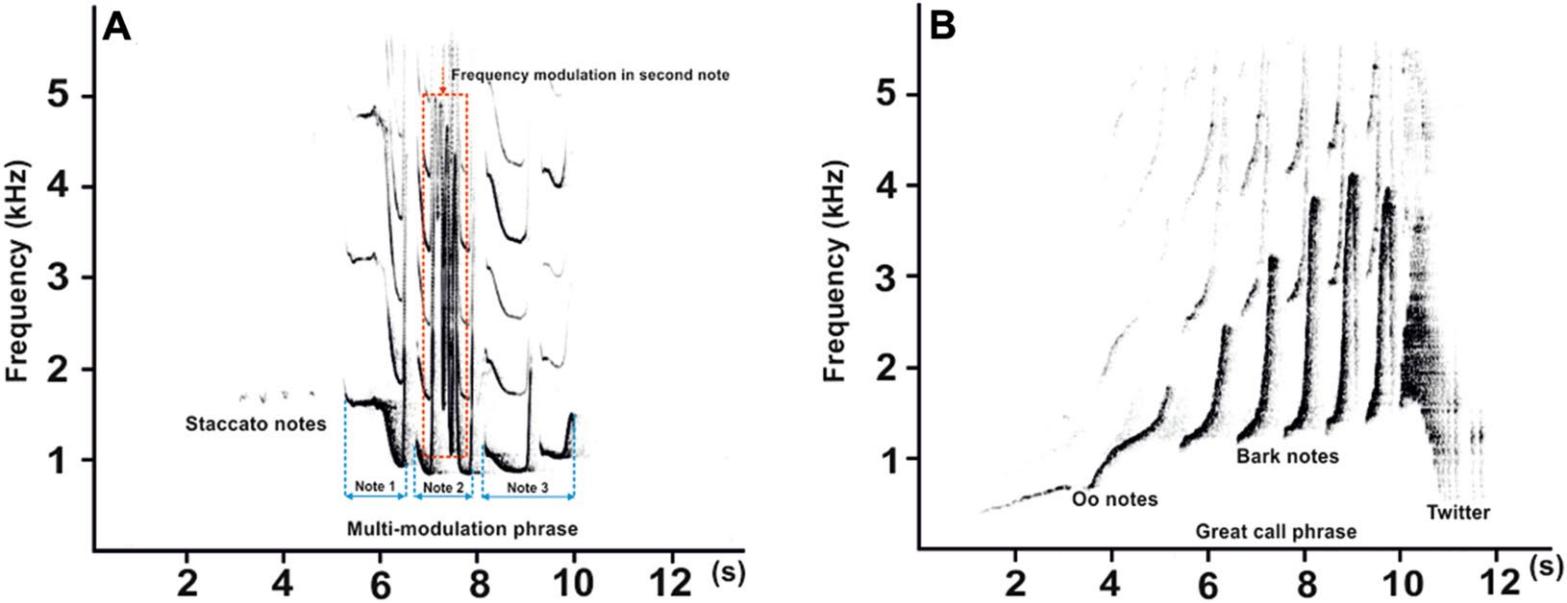
Representative spectrograms for a male (**A**) and a female (**B**) showing the vocal patterns of southern yellow-cheeked gibbons (adapted from Hradec and colleagues^20^). In the male spectrogram, the “multi-modulation” phrase is divided into three notes (blue dashed lines). The second note shows the most rapid change in frequency modulation of the steep up-and-down sweeping sound of the “multi-modulation” phrase (red dashed rectangle).

Among non-human primates, gibbons (family Hylobatidae) are a uniform group of territorial and pair-living apes that are well known for emitting vocalizations specific for species and sex^13^. For most gibbon species, paired individuals combine their respective songs into well-coordinated duets, whereas only unpaired individuals appear to produce solo songs^14^. In southern yellow-cheeked gibbons (*Nomascus gabriellae*), a full developed (mature form) male call is characterized by the presence of staccato notes and the occurrence of two or more frequency modulations in the second note of the multi-modulation phrase (Fig. 1A). The staccato notes are very subtle and uttered in short, irregular series. The multi-modulation phrase is the predominant acoustic structure. It has a maximum frequency up to 5 kHz and is further divided into three notes^15^. The adult southern yellow-cheeked gibbon male has extremely rapid frequency modulation alternation sweeps in the second note of the multi-modulation phrase that set them apart from other species of the genus *Nomascus*^13,15,16^. Whereas the vocal pattern of the male gibbon is relatively well-documented, nothing is known about the ontogeny of the pattern.

Females produce the great call^17^, which consists of so-called “oo” notes, “bark” notes and a twitter sound (Fig. 1B). The "great call" was originally thought to have been produced only by females. More recently it has been documented, however, that immature *Hylobates* and *Nomascus* males produce great calls during co-singing interactions with their mothers both in the wild and in captivity, and this occurs, for the most part, until the young male reaches five years of age^18–21^.

No information regarding the transition from the female-like great call to the male-specific call during ontogeny has been published. This transition, along with the subsequent development of the male vocal pattern, might be triggered at around 5 years of age, when physical and sexual maturation signs, which continue until at least 7 years of age, are recognized^22,23^. It is likely that the male call develops gradually due to not only maturation (e.g., physical, sexual and social) but also the more complicated vocal structure of the male call (i. e., staccato notes and the multi-modulation phrase), which is not as stereotypical as a female great call^15^. Furhermore, it is unknown if young males emit the female-like vocalization and the male calls during the same time period or if the vocal repertoire changes abruptly.

This study is the first of its kind to describe the transition from the female-like great call to the male call, as well as the male vocal development, that takes place in young male southern yellow-cheeked gibbons between the ages of 5.6–8.11 years.

We predicted that this transition as well as the development of the male call is a normal part of the aging process. If this is the case, the following phenomena will occur: a) female vocalizations are no longer produced with the mature form of the multi-modulation phrase and b) all stages of the male vocalization occur gradually as the young male ages.

## Materials and methods

### Statement on ethical standards

The research conducted herein was approved by the Ethics and Animal Care Committee at the Czech University of Life Sciences, Prague (reference number: CZU/1606), and was performed in accordance with relevant the ARRIVE guidelines (https://arriveguidelines.org). This study was fully non-invasive and approved by the management of both the Jihlava and Bojnice zoos. Both zoological institutions employ rigorous standards for animal welfare, and are accredited by the EAZA (European Association of Zoos and Aquaria) and UCSZOO (Union of Czech and Slovak Zoos). This study fully complied with the legal requirements of the Czech Republic and the Slovak Republic as well as those provided by the European Directive 2010/63/EU.

### Subjects

This study was conducted in Czech and Slovak zoological parks (two gibbon groups from the Jihlava zoo; one gibbon group from the Bojnice zoo) and involved four southern yellow-cheeked gibbon males (Table 1). All monitored young males were born in captivity. One group from the Jihlava zoo as well as the group from the Bojnice zoo comprised one adult male, one adult female, and their offspring. The other Jihlava zoo group comprised one adult female and her two-male offspring of different ages. The adult male died in 2009 when young male no. 1 was two years old and young male no. 2 was an infant aged only a few months. Young male no. 1 was nearly 9 years old when he was sent to another zoo (Novosibirsk zoological park, Russia), where he wasn’t observed. Both Jihlava zoo groups remained in visual and auditory contact with each other. Each group had permanent access to an indoor and outdoor enclosure. Both the indoor enclosures (Bojnice: 18.7 m^2^, height 4 m; Jihlava group one: 21 m^2^, height 7 m; Jihlava group two: 20 m^2^, height 7 m) and outdoor enclosures (Bojnice: 63 m^2^, height 6 m; Jihlava group one: 104 m^2^, height 13 m; Jihlava group two: 100 m^2^, height 13 m) featured platforms at various heights, trees and extensive rope systems. The outdoor enclosures at both zoos were covered with wire-mesh. The gibbons were fed four times daily with a diet consisting of fruits, vegetables, seeds, leaves, cereals and eggs. Water was available ad libitum.

**Table 1:** Overview of southern yellow-cheeked gibbon males and the composition of the family groups.

General information								
Zoo	Jihlava						Bojnice	
**No. group**	1				2		3	
**Subjects of the study**	Young male 1		Young male 2		Young male 3		Young male 4	
**Date and place of birth**	29 November 2007, Jihlava		14 October 2009, Jihlava		2 April 2011, Jihlava		2 April 2008, Bojnice	
**Age of the young males during the study**	6.5–8.11		7–7.9		5.6–6.3		6.5–8.11	
	Date, number of solo songs and number of male calls recorded	Female-like great call produced simultaneously with male call	Date, number of solo songs and number of male calls recorded	Female-like great call produced simultaneously with male call	Date, number of solo songs and number of male calls recorded	Female-like great call produced simultaneously with male call	Date, number of solo songs and male calls recorded	Female-like great call produced simultaneously with male call
**Observation calls**	28 May 2014 (6.5 years); 1; 7	Yes	19–20 October 2016 (7 years); 4; 39	Yes	19 October 2016 (5.6 years); 1; 11	Yes	16, 18 September 2014 (6.5 years); 5; 55	Yes
	6–7 November 2014 (6.11 years); 4; 69	Yes						
	20–21 May 2015 (7.5 years); 3; 50	No	15 November 2016 (7.1 years); 2; 19	Yes	15 November 2016 (5.7 years); 2; 34	Yes	18–20 June 2015 (7.2 years); 9; 106	No
	20–22 August 2015 (7.8 years); 7; 139	No						
	19–20 October 2016 (8.10 years); 2; 36	No	4 July 2017 (7.9 years); 2; 20	No	5 July 2017 (6.3 years); 2; 52	Yes	30–31 March 2017 (8.11 years); 4; 37	No
	15 November 2016 (8.11 years); 1; 12	No						
**Total number of solo songs of male call**	18		8		5		18	
**Total number of young male calls**	313		78		97		198	

### Data collection and acoustic analysis

**Figure 2:**
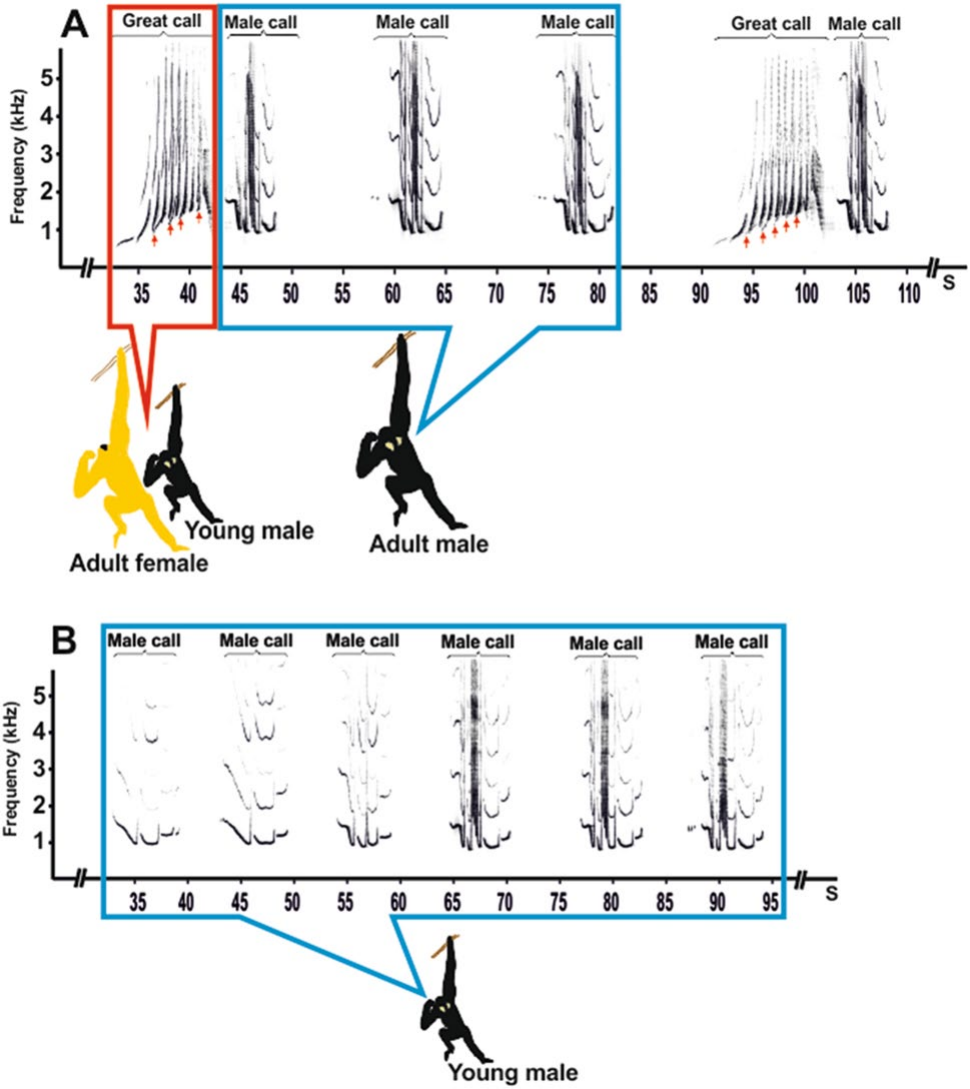
Representative spectrogram indicating the following: (**A)** duet bouts between an adult male and an adult female, with an offspring that produces a female-like “great call”; and (**B)** a young male emits a male call as a solo song after duet bouts. The red arrows indicate female-like "great calls" emitted by young males in co-singing interactions with their mothers.

This research was part of a long-term study focusing on the vocal behaviour of captive *Nomascus* gibbons. Acoustic data were collected during 3 and 6 visitations that took place between 2014 and 2017. The young males ranged from 5.6 to 8.11 years of age during the observation period (Table 1). Young males were observed for 1 to 3 days during each visitation. Spontaneous vocalizations between parents (duets bouts) were typically produced in the morning (5:00 to10:00 a.m.) and lasted approximately 15–30 min. A complete duet bout involves the the coordination of a female call (great call) and male call (staccato notes and multi-modulation phrase) (Fig. 2A). When an adult female started her great call, the adult male ceases his song and, once the female completes her great call, responded with a powerful multi-modulation phrase. He then continued to repeat several male call patterns until the female starts her next great call^15^. When the females emitted a great call, the young males followed suit and a co-singing interactions ensued in the form of a duet bout. Each duet bout was followed by male-specific vocalizations produced by the young males in the form of a solo song, which lasted 8–15 min (Fig. 2B). Vocalizations were recorded between 5:00 and 11:00 a.m. at a distance of 2 to 10 m. The vocalizations were captured on a Marantz PMD 660 recorder with a Rode NTG-2 semi-directional microphone. The sounds were recorded in mono at 16-bit resolution and with a 44.1 kHz sampling rate. All recordings were saved as waveform audio files. Sampling frequency was reduced from 44.1 to 12 kHz for adequate frequency resolution. Acoustic analysis was carried out using Avisoft SASLab Pro version 5.2 software (Avisoft Bioacoustics, Berlin, Germany). Spectrograms were generated under the following settings: FFT length = 1024; frequency resolution = 12 Hz; temporal resolution = 21.3 ms; overlap = 75%; window type = Hamming.

**Figure 3:**
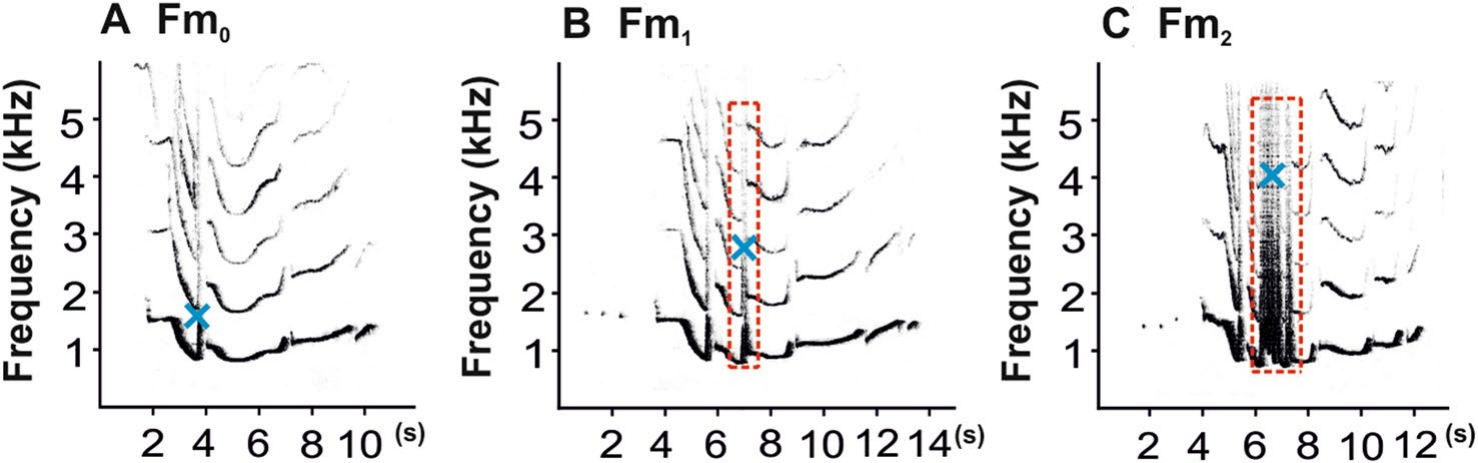
Representative spectrograms show three (**A-C**) different forms of the multi-modulation phrase in young males according to the number of frequency modulations (Fm_0-2_) in the second note (red dashed rectangle), with or without staccato notes during male vocal development. Blue crosses indicate maximum frequency.

In order to characterize the production of female-like great calls during development of the male call, we determined co-singing interactions between the mother and her young males (call with the mother) as the default acoustic parameter. With the aim of described the development of the complete male-specific vocal pattern (staccato notes and multi-modulated phrase) of southern yellow-cheeked gibbons, we analyzed only on the presence or absence of staccato notes due to their considerable individuality of expression during development. We further divided the multi-modulation phrase into three different forms (Fig. 3A-C) according to the number of frequency modulations of the steep up-and-down sweep in the second note^15^: (1) the number of frequency modulations is 0 (Fm_0_); (2) the number of frequency modulations is 1 (Fm_1_); and (3) the number of frequency modulations is 2 or more (Fm_2_). Fm_0-1_ is a non-mature form of the multi-modulation phrase. Fm_2_ is a mature form of a multi-modulation phrase. We also measured a higher pitch vocal element (kHz), i.e. maximum frequency, for each type of multi-modulation phrase.

### Statistical analysis

All data (see supplementary information file 1) were analysed using an SAS System version 9.4 (SAS Institute Inc.). The goodness of fit of each model (homoscedasticity, normality of errors and independence) was checked by visually inspecting residuals using plots = Pearson panel and testing residuals for normality by Kenward-Roger test. Where appropriate, we applied log transformation to improve normality of data distribution. Results with a p-value of less than 0.05 (*P* ≤ 0.05) were considered statistically significant. We applied a multivariate General Linear Mixed Model (GLMM, PROC GLIMMIX) for binary distribution. In order to test the transition from the female-like call to the male call during the vocal ontogeny, the model was designed with a “call with the mother” as a categorical dependent variable (yes/no). The fixed effects comprised the log-transformed age of the young males (5.6 to 8.11 years of age) and three forms of log-transformed multi-modulation phrases according to the number of frequency modulations (Fm_0-2_). To test the development of the male call, we used a GLMM with “Fm_2_” as a dependent variable. The fixed effects comprised the log-transformed age of the young males and the log-transformed maximum frequency of the multi-modulation phrase. When using the best GLMM model, we followed “Fit Statistics,” providing the best results with “age” as the only significant fixed effect.

## Results

### The transition from female-like great call to male-specific call during vocal ontogeny

**Figure 4:**
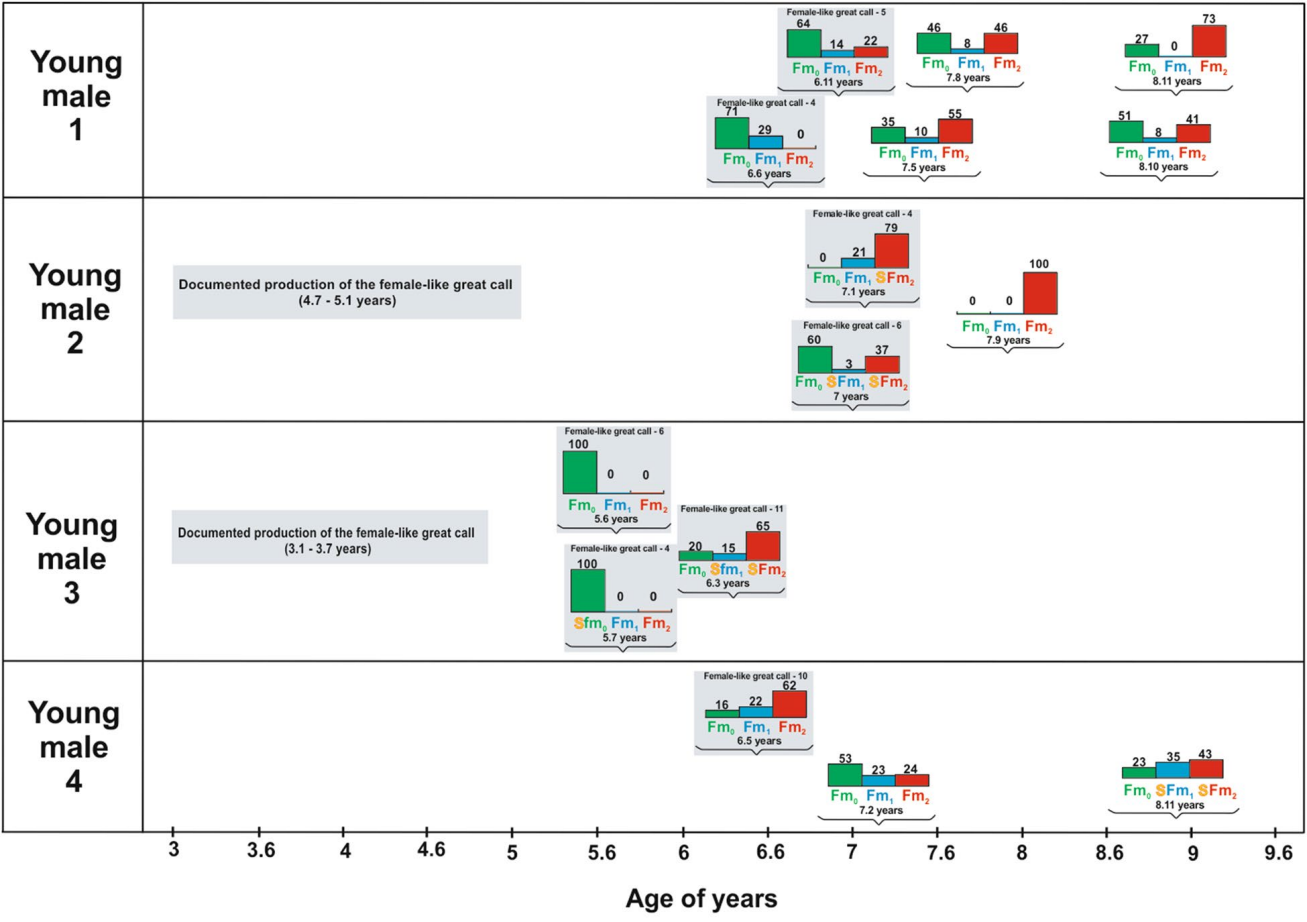
Barplots showing three different forms of multi-modulation phrases according to the number of frequency modulations (Fm_0-2_), with or without the occurrence of staccato notes (S) during male vocal pattern development. Barplots delimited by a rectangle (purple background) indicate males that continued producing female-like great calls with their mothers during male call development.

**Figure 5:**
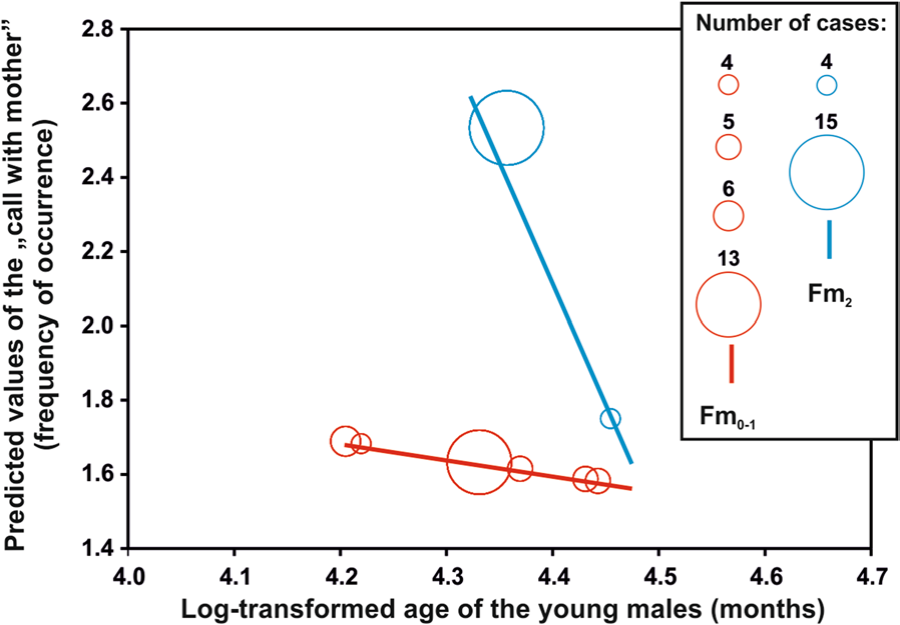
Predicted values of the “call with mother” (frequency of occurrence), i.e. production of female-like great calls by young males in co-singing interactions with their mothers plotted against the log-transformed age (months) of young males. Values for each cases are categorized according to the following forms of the multi-modulation phrase: Fm_0-1_—a non-mature form of the multi-modulation phrase, which contains one or no frequency modulation in the second note of the multi-modulation phrase; and Fm_2_—a mature form of the multi-modulation phrase, which contains two or more frequency modulations in the second note of the multi-modulation phrase.

We found that the vocal repertoire transition from the female-like great call to the male-specific call occurred gradually in the young males after they had reached 5 years of age. An unexpected finding was that the young males emitted both the female-like great call and the male-specific call during vocal ontogeny, specifically from 5.6 to 7.1 years of age (Fig. 4). Young males from 5.6 to 7.1 years of age emitted the female-like great call only during co-singing interactions with their mothers. Once they reached 7.1 years of age, the young males stopped producing female-like great calls in co-singing interactions with their mother and emitted only male-specific calls. Contrarily, young males from 5.6 to 8.11 years of age emitted the male-specific vocalization only as a solo song following vocalizations from their parents or a neighbouring group (Table 1). We found that the young males emitted the female-like great call exclusively during periods when they produced non-mature forms of a multi-modulation phrase (Fm_0,1_—none or one frequency modulation in second notes). The number of female-like great calls emitted by the young males decreased significantly with the onset of the mature form of the multi-modulation phrase (Fm_2_—two or more frequency modulations in the second note), which developed with age (F_(2,57)_ = 2.67, *P* < 0.0001, Fig. 5).

### Development of the male-specific vocalizations during ontogeny, with a focus on the staccato notes and the fully developed multi-modulation phrase

**Figure 6:**
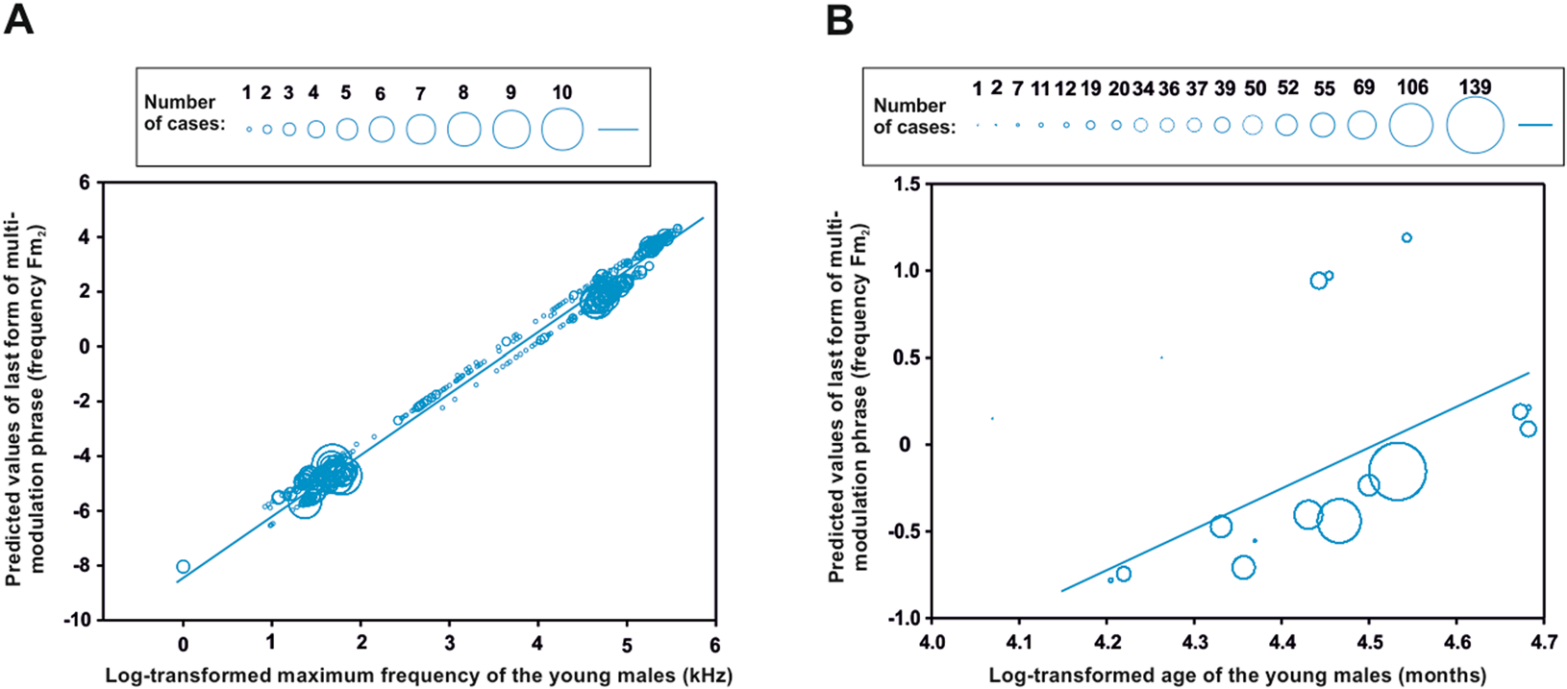
Charts showing development of the male vocal pattern: (**A**) predicted values of the last form of a multi-modulation phrase (frequency Fm_2_) plotted against the log-transformed maximum frequency of calls emitted by the young males; and (**B**) predicted values of the last form of a multi-modulation phrase (frequency Fm_2_) plotted against the log-transformed age (months) of the young males. Values for each cases indicate the number of frequency modulations (two or more) in the second note of a mature form of the multi-modulation phrase (Fm_2_).

We did not expect the multi-modulation phrase to develop as the first stage of the male vocalization. Initially, the male vocal pattern emerged through simple forms of a multi-modulation phrase (Fig. 3A) without frequency modulation, followed by the onset of a second multi-modulation phrase exhibiting the first frequency modulation (Fig. 3B), and finally with two or more frequency modulations (Fig. 3C). As the maximum frequency (F_(1, 586.9)_ = 140.59, *P* < 0.0001, Fig. 6A) and age of the young males (F_(1, 687)_ = 7.00, *P* < 0.0001, Fig. 6B) increased, so did the number of the frequency modulations of the multi-modulation phrase. Later, "staccato" notes began to develop in some young males (young males 2–4, Fig. 4). The young males began emitting the staccato notes at various ages, ranging from 67 months (young male 3) through 84 months (young male 2) to 107 months (young male 4). The first young male did not emit staccato notes at all during the observation period; he was 107 months old at the end of the recording, at which time he was sent to another zoo where he could no longer be observed (Fig. 4).

## Discussion

Our study revealed that the transition from the female-like great call to the male-specific call occurs gradually in young male southern yellow-cheeked gibbons after they reach 5 years of age, and young males aged 5.6 to 7.1 years old regularly emit both calls (female-like great calls and male-specific calls). However, after reaching 7.1 years of age, the young males emitted the male calls exclusively, and they continued to do so until the end of the observation (at 8.11 years of age).

### The change from female-like call to male call during vocal ontogeny

As we predicted, the decrease in the number of female-like great calls was attributed to the development of the mature form of the multi-modulation phrase (Fm_2_), which is considered as an indicator of the achievement of vocal and, perhaps, physical maturity^22,23^. Our study showed that the number of co-singing interactions between a young male offspring and its mother decreased significantly as the young male aged, which could have been a consequence of increased social independence, as was demonstrated in one study on the vocal development of agile female gibbons^24^. After the age of 7.1 years, the young males employed the male calls exclusively, and this continued until they reached the age of 8.11 years, when our observations ceased. Our results are in agreement with those of earlier studies on wild gibbons, which suggest that offspring remain with their families until about 8–9 years of age, at which time the males leave the natal group to search for mates and their own territory^25,26^.

To date, the production of the female-like “great call” by juvenile and adolescent male gibbons in co-singing interactions in with their mothers has been mentioned, albeit without detailed investigation, in three species, namely white-handed gibbons (*Hylobates lar*^19^), agile gibbons (*Hylobates agilis*^19^) and northern white-cheeked gibbons (*Nomascus leucogenys*^18,20^). However, those studies covered only the period from 2.11 to 5 years of age. It is likely that simultaneous vocalizations of male and female calls would also occur in white-handed gibbons, agile gibbons and northern white-cheeked gibbons. Young male southern yellow-cheeked gibbons produce female-like great calls from about 2.3 to 5 years of age^21^. Moreover, our results show that young males produce the female-like great call until they reach 7 years of age. This suggests that, during male vocal ontogeny, the boundaries separating the vocal repertoires of males and females are not as strictly defined as one would expect considering the predominantly innate structure of gibbon songs^27,28^.

The question remains as to the purpose of the co-singing interactions between young males and their mother, which take place until the offspring reach 7 years of age. Without proper data and hormone analysis, we can only speculate on this vocal behaviour. In our previous study, it has been suggested that immature southern yellow-cheeked gibbon males up to the age of 5 most likely emit the female-like great call in order to strengthen the family bond and/or relay information about the immature status of the offspring^21^. It is possible that males over 5 years of age (i.e., after reaching sexual maturation) continue emitting both calls (i.e., female-like great calls and male calls) in order to maintain social cohesion with their mothers^29,30^ and/or pass information regarding their own individual status to the father^21^. Gibbons are territorial^31^ and when a young male emits a male call, it might trigger aggressive behaviour from a father against his maturing young males^22,23^. Additionally, it has been suggested that these co-singing interactions might serve as a kind of ‘‘trigger’’ for song training (vocal training) due to the stable vocal structure (great call) of the adult female^19^. These regular interactions might also facilitate the gradual shaping of the supralaryngeal vocal tract^32^ in the young males, making it easier for them to develop their own vocal repertoire during vocal ontogeny.

### Development of male-specific calls in southern yellow-cheeked gibbons

We did not expect to see the multi-modulation phrase develop gradually and the staccato notes develop in leaps. In previous studies, the vocal patterns in adult males have been described as one vocal pattern alternating between staccato notes and multi-modulation phrases, usually in that order^13,15^. However, our study surprisingly revealed that staccato notes and multi-modulation phrases do not evolve at the same time and their occurrence was not mutually dependent. The multi-modulation phrase developed first and later, in certain young males (young males 2–4) appeared staccato notes. An individual-specific time delay occurred between the development of staccato notes and multi-modulation phrase.

Our results indicate that "staccato" notes, which could support flexible production in an irregular series, are less of a fixed part of the male vocal repertoire. Huang and colleagues^33^ reported that paired adult male Cao-vit gibbons (*Nomascus nasutus*) and western black-crested gibbons (*Nomascus concolor*) produced a higher quantity of staccato notes than did unpaired males. Because our observations did not include gibbon males over 8.11 years of age, it is very likely that staccato notes may develop later in ontogeny.

In this study, the development of all three forms of the multi-modulation phrase (Fm_0-2_) was based on an increase in the number of frequency modulations and was related to age and maximum frequency, with the last form of multi-modulation phrase (Fm_2_) prevailing in the later stages of ontogeny. However, it seems that the frequency modulation, consisting of a steep up-and-down sweeping of the second note of the multi-modulation phrase, is much more flexible than the overall pattern of the multi-modulation phrase.

Moreover, of all the gibbons of the genus *Nomascus*, adult male southern yellow-cheeked gibbons emit the fastest frequency modulation in the second note of the multi-modulation phrase, which is reminiscent of trills in birds such as those of the Emberizidae family^34^, the banded wren (*Thryothorus pleurostictus*^35,36^) or in mammals such as neotropical singing mice (the genus Scotinomys^37^). For most of these species, a trill with higher vocal properties may be a reliable indicator of the caller´s physical condition, which is mostly affected by androgen levels^35,37,38^.

In addition, higher levels of androgens are strongly correlated with higher pitched vocal elements (e.g. maximum frequency) in white-handed gibbon males^39^. It is highly probable that high androgen levels contribute to larynx growth (changes in laryngeal muscle) during development of the male call; androgen receptors are located on the laryngeal cartilages^40^, which create tension in the the vocal folds (controlled by the cricothyroid muscle), leading to the production of the high-frequency call^41^. Based on these morphological and physiological changes, the female-like features of the great call can slowly diminish, allowing full development of a mature form of the multi-modulation phrase (Fm_2_), which is typical in adult males.

We are fully aware of the small sample size of this descriptive study. However, such vocal development has never been recorded in any males of a non-human primate species, including prosimians^3^, new world monkeys (e.g.^5,7,8,42^), old world monkeys (e.g.^10–12,43^) and great apes (e.g.^44,45^).

## Conclusion

Our study is the first of its kind to provide evidence that young male southern yellow-cheeked gibbons regularly emit both female-like great calls and male-specific calls between the ages of 5.6 to 7.1 years. The change in the vocal repertoire was not an abrupt one but, rather, a gradual acquisition of a male-specific call, which continued to at least 8.11 years of age.

Future experiments are required in order to clarify whether androgen levels affect vocal ontogeny in male offspring, with an emphasis on the overlap between both types of calls. Given that the family *Hylobatidae* consists of four genera and approximately 20 primate species, further comparative research is highly desirable. Vocal ontogeny remains an unresolved issue that needs to be explored further in order to have a thorough understanding of the vocalizations of these amazing singing apes.

## Supplementary Information

Supplementary Information.

## Data Availability

The datasets analyzed during the current study are included as Supplementary information file 1. Access to raw sound files will be provided upon reasonable request to the corresponding author.
